# Monkey Do, Monkey See? The Effect of Imitation Strategies on Visuospatial Perspective-Taking and Self-Reported Social Cognitive Skills

**DOI:** 10.3390/bs15081112

**Published:** 2025-08-17

**Authors:** Marion Ducret, Eric Chabanat, Ayumi Kambara, Yves Rossetti, Francois Quesque

**Affiliations:** 1 Inserm UMR-S 1028, CNRS UMR 5292, Equipe Trajectoires, Centre de Recherche en Neurosciences de Lyon, 69676 Bron, France; 2 Department of Humanities, Faculty of Psychology, Kyoto University of Advanced Science, Kyoto 616-8361, Japan; 3 Plateforme “Mouvement et Handicap” and Plateforme NeuroImmersion, Hôpital Henry-Gabrielle, Hospices Civils de Lyon, 20 Route de Vourles, 69204 Saint-Genis-Laval, France; 4 Laboratoire Sur Les Interactions Cognition, Action, Émotion (LICAE), Université Paris Nanterre, 200 Avenue de La République, 92001 Nanterre, France; 5 Centre Ressource de Réhabilitation Psychosociale, CH Le Vinatier, 69678 Bron, France

**Keywords:** visuospatial perspective-taking, social cognition, imitation, motor representation, embodied cognition

## Abstract

Classical social cognitive conceptions suppose that the existence of common representations between agents constitutes the basis that represents the world from others’ perspectives. Alternatively, recent contributions support that the ability to distinguish self- from other’s representation would rather be at the origins of social inferences abilities. In the present study we compared the effects of two types of imitation training: mirror imitation (for which gesture could be represented in common referential) and anatomically congruent imitation (which requires not only a representation of the gesture of the model but also distinguishing between one’s own and others’ representations). We observed that a 4 min training of anatomically congruent imitation, but not of mirror imitation, improved performance on a visual perspective-taking test. This short training did not significantly impact self-reported measures of social cognitive skills. These results suggest that a unique transversal cognitive mechanism of co-representing and switching between self-related and other-related representations could be involved at both the motor and the mental-state levels. Opportunities for innovative social cognitive interventions at the motor level are discussed.

## 1. Introduction

Humans spend most of their time interacting with congeners. In this context, they have developed numerous abilities to assure fluid and efficient social interactions, classically referred to as “social cognition” (e.g., Gallotti & Frith, 2013). Among the vast range of social cognitive abilities (from face perception and gaze detection to vicarious learning and mentalizing), crucial skills enable us to anticipate others’ behaviors, such as the ability to infer others’ perspectives on the world. Over the last decades, most of the attention in the field of social cognition has focused on shared representations. The existence of a mirror system in humans, composed of neurons that fire to both the execution and the observation of specific types of actions, may provide a common neural code for motor and perceptual processes (Fox et al., 2016). In this context, it has been conceived that spontaneous motor simulation of the action of others engenders the experience of what they physically experience. This concept was foundational to the development of embodied approaches to social cognition, which argue that shared sensorimotor representations play a causal role in self–other understanding, placing the body at the core of the processing of others’ mental states (de Vignemont, 2014; Keysers & Gazzola, 2009; Quesque & Coello, 2015; Tsakiris, 2017). The existence of common motor representations between self and others would serve as the basis for forming more abstract representations of the world from others’ perspectives (Gallese & Goldman, 1998; Niedenthal, 2007; Rizzolatti & Craighero, 2004). According to this hypothesis, imitation should facilitate the ability to represent others’ perspectives, as the person imitating and the one being imitated would temporarily share common representations.

All types of inferences about others’ mental states require an ability to represent mental states that differ from what is directly experienced in the here and now (Epley & Caruso, 2009; Erle & Topolinski, 2017; Quesque & Rossetti, 2020), overcoming our natural egocentric vision of the world. Sharing a common representation might then even interfere with the ability to attribute mental representation to other agents in the case of a mismatch between self and other representations (see Saxe, 2005). In this context, and contrasting the mirror system hypothesis, it has been suggested that the cognitive mechanism allowing representations pertaining to the self and to others to be distinguished would rather constitute the foundation of higher-order social inferences (Brass et al., 2009). The ability to discriminate mental representations based on their origin would evolve from the motor ability to distinguish between self-generated actions and actions controlled by external sources (Brass & Heyes, 2005; Quesque & Brass, 2019; Jeannerod & Pacherie, 2004). According to this view, imitation should interfere with the ability to infer others’ mental representations by temporarily blurring the frontiers between one’s own and other’s motor actions.

Following this reasoning, Santiesteban et al. (2012) used “imitation” and “imitation-inhibition” training to, respectively, decrease and increase the level of self–other control and examined the resulting effects on visuospatial perspective-taking (i.e., “the process by which one represents a scene from another person’s viewpoint, by adopting their perspective”; Quesque et al., 2024). In the “imitation” condition, participants were presented with stimuli depicting hand movement (e.g., index finger lifting) and had to produce the same action, whereas in the anti-imitation condition, they had to inhibit the motor representation generated by the observation of the stimuli and produce an alternative action (e.g., lift their middle finger). Participants trained to inhibit their tendency to imitate showed better performance on the visual perspective-taking task compared to participants trained to imitate or even to control participants who received a general—non-motor—inhibition training (no differences were reported between the latter two conditions), supporting the idea that the gain is specifically related to the management of self- and other-related representations rather than a general improvement in conflict resolution abilities. Using the same paradigm, de Guzman et al. (2016) observed an increase in self-reported “empathy” (note that recent collaborative efforts for a unified terminology in social cognition might favor more specific lexical alternatives such as “mentalizing” and “empathic concern”; Quesque et al., 2024) for their participants following an “imitation inhibition” training compared with an “imitation” training.

A crucial point about imitation is however rarely addressed in cognitive science research: there are several reference frames in which we can imitate each other. In the experiments described above, the stimuli used are very minimal (e.g., classically, only one vertically oriented hand is presented to participants; see Cracco et al., 2018, for a review) in order to avoid effector-matching problems. However, when it comes to designing intervention techniques that can be easily implemented in clinical settings, it is particularly relevant to use face-to-face imitation games involving large-scale upper limb movements (McGarry & Russo, 2011; Coutté et al., 2024). In the present study, we aim to bridge the gap between highly constrained, lab-designed procedures and applied interventions, which often lack critical reflection on the type of imitation and movement employed.

Shield and Meier (2018) identified four possible strategies for imitating. Imitation may be performed in anatomical coordinates, i.e., by activating the exact same muscles as the model. It may be achieved by mirroring the model. Visual strategies may also be adopted. It may consist in reproducing what I see from my own perspective. And it may finally consist in reproducing what the model would see from their perspective. Several sources of data agree that imitation is most spontaneously performed as mirroring, both in adults and children (e.g., Wapner & Cirillo, 1968; Chiavarino et al., 2007; Franz et al., 2007; Shield & Meier, 2018)^1^. Mirroring is even increased in patients with frontal lobe lesion (e.g., Lhermitte et al., 1986) or with autism spectrum disorder (Shield & Meier, 2018), and this may be related to inhibitory processes or to a deficit for remembering appropriate stimulus–response matching (Chiavarino et al., 2007), as it was originally suggested by Bekkering et al.’s (2000) study on imitation in children.

Building directly on the work of Santiesteban et al. (2012) and de Guzman et al. (2016), we aimed to compare the effects of two imitation strategies (anatomically congruent imitation and mirror imitation) on a visuospatial perspective-taking performance task and on self-reported measures of social cognitive skills. Because the mirror strategy appears to be the default mode of imitation, we predicted that using this mode would be easier than the anatomical mode. However, since anatomically congruent imitation—unlike mirror imitation—requires dealing with common motor representations and inhibiting spontaneous mirroring responses, we expected participants to perform better on social cognitive tasks requiring self–other distinction after being trained in anatomically congruent imitation (compared to mirror imitation).

## 2. Materials and Methods

### 2.1. Participants

A total of 76 adults (37 women, age range 18–28 years, M = 21.51, SD = 1.97) participated in this study. Participants were randomly assigned to the mirror imitation (N = 38) or anatomically congruent imitation (N = 38) groups. Groups did not differ in terms of age (t(74) = 0.4, *p* = 0.69) or gender balance (χ^2^(1) = 0.05, *p* = 0.82). The sample size was relatively arbitrary, as experimenters were instructed to recruit as many participants as possible for a given period. Importantly, our sample size is two times bigger than the ones of de Guzman et al. (2016) and Santiesteban et al. (2012). Moreover, sensitivity power analyses conducted with GPower (1−β = 0.80, α = 0.05, two-tailed) allowed us to identify a partial Eta-squared (ηp^2^) effect size of 0.0102 (which is classically conceived to be small, Cohen, 1988) with a probability of 0.80 considering our current experimental design and sample (Faul et al., 2007). All participants were naïve to the research aims of this study and gave their written informed consent before taking part in the experiment. This study conformed to the principles of the revised Declaration of Helsinki (World Medical Association, 2013).

### 2.2. Procedure

Two experimenters (one male, one female) aged 19 years and naïve to the hypotheses were independently in charge of recruiting participants and running the experiment. After having given their informed consent, all participants were randomly assigned to one of the two trainings (anatomically congruent imitation; mirror imitation). After the training, participants had to perform a computerized visuospatial perspective-taking task and filled in the IRI questionnaire (Davis, 1983) in that order.

#### 2.2.1. Training

**Figure 1:**
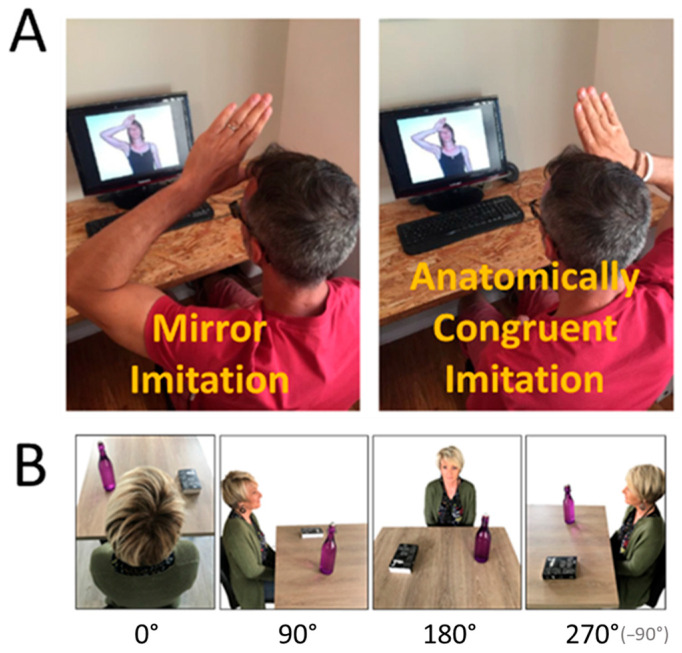
(**A**). Illustration of the “Mirror imitation” (**left**), in which the observer’s right side corresponds to the actor’s left side and of the “Anatomically congruent imitation” (**right**), in which the observer’s right side corresponds to the actor’s right side. (**B**). Illustration of the stimuli used in the computerized visuospatial perspective-taking task.

During the training, participants were presented 40 pictures representing an actor performing different manual actions (e.g., putting their left palm on the right shoulder; putting their right fist under their chin.). Each picture was presented three times, for a total of 120 stimuli displayed in a randomized order. The stimuli were presented to participants in a row, on a computer screen (15 inches), with a duration of 2000 ms per stimulus. Participants were asked to produce the gestures performed by the actor in real time. In the “Mirror imitation” group, participants were told that they should consider the pictures displayed as a mirror (e.g., if the left hand is moved by the actor, you should perform the same action with your right hand, see Figure 1A for an illustration). In the “Anatomically congruent imitation” group, participants were told that they should produce identical actions (e.g., if the left hand is moved by the actor, you should perform the same action with your left hand, see Figure 1A for an illustration). The training session last approximately 4 min. The experimenter was present during the whole training and checked whether participants successfully imitated each stimulus or not according to the instructions previously received. An objective score (max = 120/120) linked to the difficulty of the task was then computed for each participant. Participants were also asked to explicitly report how difficult they found the task using an ordinal scale ranging from 0 (not difficult at all) to 100 (extremely difficult). Finally, participants had to evaluate how “nice”, how “moral” the actor was and how “close” they felt to her using similar 100-point scales.

#### 2.2.2. Computerized Visuospatial Perspective-Taking Task

Participants had to perform a visuospatial perspective-taking task inspired by the one used by Michelon and Zacks (2006). This task relies on photographs of an actor (different from the one used in the training) seated at a table on which a bottle and a book were displayed (see Figure 1B for an illustration). The objects were, respectively, displayed at the left and right of the participants (position counterbalanced across stimuli), and these scenes were taken from 4 different perspectives (resulting in 8 different stimuli). The angular disparity between the participant’s position and the actor’s position then varied from 0° to 270° (=−90°) in increments of 90° (0° = actor’s back; 90° = actor’s left; 180° = facing the actor; 270° = actor’s right, see Figure 1B. for an illustration). The experiment was run with Matlab© software on a portable computer (script of the task is available at https://osf.io/n3wge/, accessed on 9 February 2020). Each trial started with a central white fixation cross on a black screen (500 ms), followed by one of the photographs presented for a maximal duration of 3000 ms or until participants’ response. Stimuli were displayed 6 times, resulting in 48 trials (12 per perspectives), presented in a randomized order. Participants had to judge whether a target object (e.g., the bottle) was on the actor’s left or right side. Participants were instructed to respond as fast and as accurately as possible by using two response keys labeled “Left” and “Right”. To avoid stimulus–response incompatibility, the “Left” key was on the participant’s left and the “Right” key was on the participant’s right as in Michelon and Zacks (2006). Two dependent variables were measured: response time (RT) and the number of errors.

#### 2.2.3. IRI Questionnaire

Participants then completed the Interpersonal Reactivity Index (IRI; Davis, 1983), a self-reported multidimensional measure of social cognitive abilities. The IRI is composed of four 7-item subscales (perspective-taking; fantasy; empathic concern; personal distress). For each item participants respond on 5-point Likert scale ranging from “does not describe me well” to “describes me very well”. While classically considered a trait measure, this questionnaire allowed us to estimate participants’ natural tendency to consider others’ perspectives (providing an explicit self-reported measure of mentalizing), as well as their empathic concern (allowing us to replicate the findings of de Guzman et al., 2016). A total of 5 questionnaires were lost prior to processing by one of the experimenters (3 in the mirror imitation condition, 2 in the anatomically congruent imitation condition).

### 2.3. Data Analysis

Data are available at https://osf.io/n3wge/ (accessed on 9 February 2020). For the computerized visuospatial perspective-taking task, trials were excluded from the response time analysis when a participant responded erroneously (2.8% of the data) or if response times were shorter than 300 ms or more than 3 standard deviations away from the median (2.7% of the data) computed for each participant (Leys et al., 2013). We chose to use a mixed linear model to process the response time data. This model is advantageous with respect to ANOVA since it takes into account fixed effects (the “real” effects we want to study) as well as random effects (each subject may have their own intercept in the linear relation between RT and independent variables). For categorical variables such as the condition “anatomically congruent” vs. “mirror” imitation, the fitted parameter was evaluated as the slope parameter in RT between a chosen reference (here “anatomically congruent”) and “mirror”. The orientation variable with four modalities (“back” 0°, “face” 180°, “left” 90°, and “right” 270°) as treated by choosing “back” as reference, and 3 fitted parameters were evaluated as slope parameters between “face” and “back”, “left” and “back”, and “right” and “back”. As a direct consequence, in mixed linear model, effect sizes are given by these parameters (directly expressed in ms) and their 95% confidence intervals (also expressed in ms). A floor effect was observed for accuracy performance, with participants making very few errors (typically 0, 1, or 2), resulting in a high frequency of zero scores. Due to this limited variability, particularly at the trial level, fitting a mixed-effects model with subject-level random effects would have been unreliable or prone to convergence issues. Therefore, an ANOVA was conducted with “imitation condition” as a between-subject factor and “orientation” as a within-subject factor, and effect sizes were indexed using partial Eta-squared (ηp^2^). Other between-group comparisons were conducted with standard T-tests and their respective effect size estimated using Cohen’s d and their 95% confidence intervals. Bonferroni’s correction was applied for post hoc comparisons.

## 3. Results

### 3.1. Training

During the training session, participants from the “Anatomically congruent imitation” condition produced significantly more mistakes (m1 = 10) than participants in the “Mirror imitation” condition (m2 = 3, *t*(74) = 6.62, *p* < 0.001, Cohen’s d= 1.52, 95% CI = [1, 2.03]). Congruently, participants from the “Anatomically congruent imitation” condition judged the training as more difficult (m1 = 41.1) than participants in the “Mirror imitation” condition (m2 = 19.5, *t*(74) = 5.66, *p* < 0.001, Cohen’s d = 1.03, 95% CI = [0.81, 1.8]). Finally, participants’ evaluation did not differ between the two conditions concerning how “nice” the actor was rated (m1 = 58.9, m2 = 62.3, *t*(74) = 0.77, *p* = 0.45, Cohen’s d = −0.17, 95% CI = [−0.63, 0.28]). This was also the case for the “morality” (m1 = 56, m2 = 56.1, *t*(74) = 0.04, *p* = 0.97, Cohen’s d = −0.01, 95% CI = [−0.45, 0.44]) and “closeness” judgements (m1 = 27.5, m2 = 31.6, *t*(74) = 0.76, *p* = *0*.45, Cohen’s d = −0.18, 95% CI = [−0.63, 0.28]).

### 3.2. Visuospatial Perspective-Taking Task

**Figure 2:**
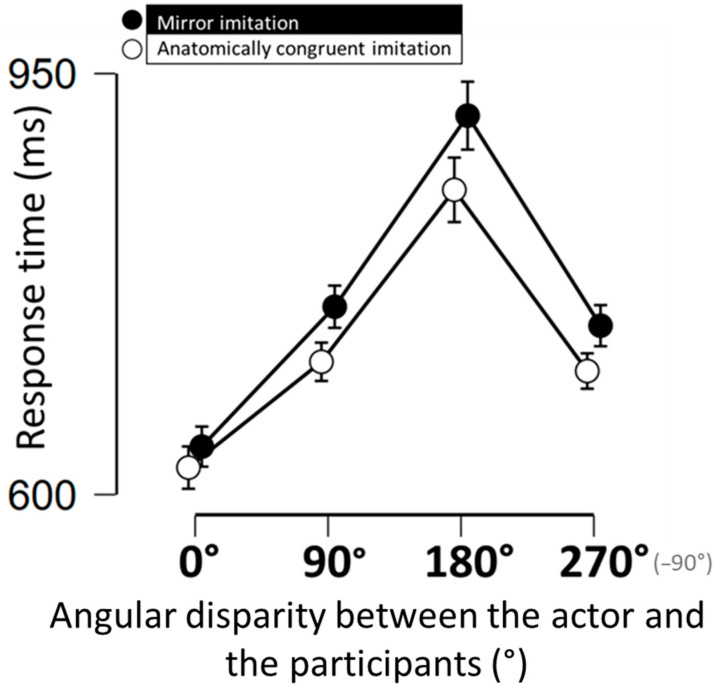
Response times (in milliseconds) in the visuospatial perspective-taking task as a function of the angular disparity between the actor’s and the participant’s orientations. Vertical bars represent confidence intervals at 95%.

Concerning response times, we observed a main effect of the angular disparity between participants’ orientation and actor’s orientation. Precisely, participants were significantly slower in the “left/90°” (diff = 98 ms, t = 4.459, *p* < 0.001, 95% CI = [25.7, 170.7]), “face/180°” (diff = 283 ms, t = 8. 9, *p* < 0.001, 95% CI = [220.9, 345.6]) and “right/270°” (diff = 109 ms, t = 4.2, *p* < 0.001, 95% CI = [36.6, 181.6]) condition than in the “back” 0° condition used as reference (m = 630, see Figure 2 for an illustration). A main effect of the type of imitation training was also observed on response times, with shorter response times in the “anatomically congruent” imitation condition (diff = 88.5 ms, t = 1.97, p < 0.0595%, CI = [0.27, 176.7]) than in the “mirror” imitation condition (see Figure 2 for an illustration). No interaction was found between the type of training received by participants and the angular disparity between the participants’ and the actor’s orientation in the visuospatial perspective-taking task. Concerning accuracy performance, differences in accuracy were observed among the different angular disparities between the participant’s and actor’s orientation (F(3,74) = 6.59, *p* < 0.001, ηp^2^ = 0.08). Precisely, post hoc comparison revealed that participants made more mistakes in the left (2.9%, *p* = 0.03, Cohen’s d = 0.33, 95% CI = [0.1, 0.56]), right (2.5%, *p =* 0.001, Cohen’s d= 0.48, 95% CI = [0.24, 0.71]) and face (4.8%, *p* = 0.003, Cohen’s d = 0.42, 95% CI= [17.9, 64.8]) orientation conditions than when they shared the same perspective as the actor (1%). No differences in accuracy were observed between the three former conditions. We found no significant effect of the imitation conditions (F(1,74) = 0,04, *p* = 0.84, ηp^2^ = 0.001). No interaction was found between the imitation trainings and the angular disparity in the visuospatial perspective -taking task (F(3,74) = 0.04, *p* < 0.77, ηp^2^ = 0.005).

### 3.3. IRI Questionnaire

Participants’ IRI scores did not differ between the “Anatomically congruent imitation” condition and the “Mirror imitation” condition (m1 = 116. and m2 = 117.5, respectively, t(69) = −0.23, *p* = 0.81, Cohen’s d = −0.06, 95% CI = [−0.52, 0.41]). Moreover, no difference were observed for any of the sub-scores (perspective-taking, m1 = 29, m2 = 31, *t*(69) = −1.41, *p* = 0.16, Cohen’s d = −0.34, 95% CI= [−0.8, 0.13]; fantasy, m1 = 30.4, m2 = 29.3, *t*(69) = 0.71, *p* = 0.48, Cohen’s d = 0.168, 95% CI = [−0.3, 0.63]; empathic concern, m1 = 32.4, m2 = 31.8, *t*(69) = 0.41, *p* = 0.69, Cohen’s d = 0.1, 95% CI = [−0.37, 0.56]; personal distress, m1 = 24.9, m2 = 25.5, *t*(69) = 0.39, *p* = 0.7, Cohen’s d = −0.09, 95% CI = [−0.56, 0.37]).

## 4. Discussion

Recent experiments suggested that anti-imitation training can enhance social cognitive processes (e.g., de Guzman et al., 2016; Santiesteban et al., 2012), while imitation training does not lead to any significant benefit. We here state that imitation should not be conceived as a unitary process, and we contrasted the effects of two imitation strategies (anatomically congruent imitation and mirror imitation) on a visuospatial perspective taking-task and using self-reported measures of social cognitive skills. Specifically, we expected participants to show increased performance on the social cognitive tasks after receiving training on anatomically congruent imitation (as compared to mirror imitation), as previously reported for anti-imitation training in previous studies.

Our study confirmed the previous finding that “Anatomically congruent imitation” is more difficult than “Mirror imitation” (e.g., Wapner & Cirillo, 1968). This is supported by both explicit reports from the participants and objective measures (for example, the number of mistakes was substantially larger in the “Anatomically congruent imitation” than in the “Mirror imitation” condition). This difference in difficulty might be partly explained by spatial compatibility: mirror imitation corresponds to a spatially compatible response, whereas anatomically congruent imitation is spatially incompatible. This spatial mismatch likely increases cognitive demands, as suggested by the seminal work of Simon and Rudell (1967), requiring participants to inhibit more dominant responses. In spite of this more effortful training, participants in the “Anatomically congruent imitation” condition subsequently exhibited significantly better performance (response times shorter by 88.5 ms) on the visuospatial perspective-taking task than participants in the “Mirror imitation” condition. Overall performance on this task was congruent with previous results in the literature (Kessler & Rutherford, 2010; Kessler & Thomson, 2010; Michelon & Zacks, 2006; Muto et al., 2018). Specifically, the amount of time needed to represent the actor’s perspectives was positively linked to the angular disparity between the participants and the actor. Interestingly the proportion of errors also increased when participants had to take a perspective that differed from their own, revealing a cost for visuospatial perspective-taking judgments. The difference in visuospatial perspective-taking performance cannot be explained by an effect of fatigue as the “Anatomically congruent imitation” training was more difficult than the “Mirror imitation” training. This difference was also not the consequence of negative associations between the actors in the training and the visuospatial perspective-taking task. First, different actors were used in the two tasks. Second, no between-group difference was observed in participants’ explicit evaluation of the actors. This second point is congruent with previous findings reporting that affective judgement towards a target person and the tendency to take that person’s visuospatial perspective are independent variables (Quesque et al., 2018; Quesque et al., 2021).

The fact that participants exhibited better visuospatial perspective-taking performance in the “Anatomically congruent” than in the “Mirror imitation” condition is in line with the rationale developed by Santiesteban et al. (2012). In their study, they showed that “anti-imitation” (opposed to “imitation”) training increased visuospatial perspective-taking performance. According to their promising hypothesis, imitation-inhibition training promotes processes that distinguish and control representations pertaining to the self and the other, which constitute the foundations of perspective-taking abilities. Our results remain compatible with this general interpretation but also support that different types of imitation should be contrasted. When imitation is performed in anatomical coordinates, participants not only need to represent the gesture of the model but also deal with one’s own and others’ body representations. When simply mirroring the model, only the gesture needs to be represented, and it can be merely represented in a self-centered referential. Overall and in accordance with Santiesteban et al. (2012), our data reinforce the claim that the ability to represent others’ mental representations is rooted in the motor ability to distinguish between self-generated actions and actions controlled by external sources (see Bardi & Brass, 2016; Quesque & Brass, 2019; Sowden & Catmur, 2015). We assume that in our anatomically congruent imitation condition, a general self–other distinction process is trained, which leads to an improvement in the ability to represent the visuospatial perspective of another person while inhibiting one’s own self-centered perspective. As a consequence, we further postulate that practicing any task that requires distinguishing between self and other representations (including certain imitation strategies) may enhance the ability to represent others’ mental states. Other schemes could then be implemented to boost participants’ social inferences abilities. At the conceptual level, some evidence is already available and supports our prediction (Keysar, 1994; Simpson & Todd, 2017; Todd et al., 2011). For example, it has been shown that participants have more difficulties in representing ingroup (low self–other distinction at the conceptual level) than outgroup (high self–other distinction at the conceptual level) visuospatial perspectives (Simpson & Todd, 2017). Our results are however incongruent with those of de Guzman et al. (2016) as we did not find any difference in terms of self-reported empathy between our two groups of participants. One option to account for this divergence is the fact that we used a different self-reporting measure, i.e., the IRI (Davis, 1983), and not the QCAE (Reniers et al., 2011). However, both measures originate from well-validated self-report questionnaires. Alternatively, and congruent with the present results, we postulate that no effect of our different training should be found on empathic reaction measures. Contrasting with perspective-taking, some empathic reactions might not require distinguishing between self and other representations, i.e., specifically when related to affect sharing (Coll et al., 2017). These empathic reactions suppose a privileged link between self and others. Along this vein, studies manipulating self–other merging reported its effect on the emotional reactions triggered by others (e.g., De Coster et al., 2013; De Coster et al., 2017).

As with any study, this work has certain limitations that should be acknowledged. While the present between-subject design follows standard practices commonly used in social and cognitive psychology, including baseline measures in future studies would offer additional control and allow the assessment of individual visuospatial and socio-cognitive abilities and the investigation of the impact of personal dispositions. Additionally, our sample consisted primarily of WEIRD (Western, educated, industrialized, rich, and democratic; Henrich et al., 2010) participants, which may limit the generalizability of our findings to broader, more diverse populations. Future studies will need to verify whether similar patterns of results are observed in other populations, who might rely on different psychological strategies (see Samuel et al., 2024, for a discussion) to perform this type of socio-cognitive task. Nevertheless, these limitations do not detract from the main contribution of this study that a 4 min training in “Anatomically congruent imitation” can significantly enhance participants’ ability to represent others’ perspectives. At the fundamental level, this study builds on previous evidence (e.g., Santiesteban et al., 2012) and provides further support of the idea that the same self–other distinction process underlies imitation control and perspective-taking (Brass et al., 2009; Brass & Heyes, 2005; Quesque & Brass, 2019; Sowden & Catmur, 2015). At a more applied level, this result could ultimately lead to the development of efficient, low cost, and easy-to-implement interventions to promote perspective-taking, e.g., in the form of children’s games or clinical rehabilitation. As a first step, it would be interesting to test the social–cognitive performance of fluent sign language users. As this population is trained in imitating gestures using an anatomically congruent strategy and might even favor this type of imitation compared to non-signers who prefer the mirroring strategy (Shield & Meier, 2018), testing their ability to take spatial perspective may reinforce the present account through more longitudinal practices.

## Data Availability

Data are available at https://osf.io/n3wge/, accessed on 9 February 2020.

## Note

1 Beyond the somehow arbitrary nature of some terms, it may be pointed out that there is an incongruency between the so called “mirror neurons” and the fact that we are more prone to mirror than to imitate in anatomical coordinates. If we were to truly imitate others, i.e., share a common sensory and motor experience, we should rather spontaneously select anatomical reference frame over the visual reference frame used in mirroring.
